# Pulmonary Lymphangitis Carcinomatosa and Acute Pancreatitis: A Rare Presentation of Choledochal Cyst

**DOI:** 10.1155/1999/76976

**Published:** 1999-04

**Authors:** D. M. Kelly, P. J. O'Donnell, E. R. Howard

**Affiliations:** 1 Department of Surgery King's College Hospital Denmark Hill London SE5 9RS United Kingdom; 2 Department of Histopathology King's College Hospital Denmark Hill London SE5 9RS United Kingdom

## Abstract

Pulmonary lymphangitis carcinomatosa is an unusal cause of death in a young adult. This case describes an apparently healthy young woman who presented with severe acute pancreatitis, which is a recognized complication of a choledochal cyst. Autopsy examination revealed advanced malignancy with poorly differentiated adenocarcinoma penetrating the wall of the choledochal cyst and metastatic adenocarcinoma in the lymph nodes, lungs and kidneys. This case emphasises the unusual presentation of a choledochal cyst with acute pancreatitis and the aggressive nature of malignancy associated with this congenital anomaly.


*HPB Surgery*, 1999, Vol. 11, pp. 163–169
Reprints available directly from the publisher
Photocopying permitted by license only

© 1999 OPA (Overseas Publishers Association) N.V.
Published by license under
the Harwood Academic Publishers imprint,
part of The Gordon and Breach Publishing Group.
Printed in Malaysia.

### Pulmonary Lymphangitis Carcinomatosa and Acute Pancreatitis: A Rare Presentation of Choledochal Cyst

D. M. KELLY[a], P. J. O'DONNELL[b] and E. R. HOWARD[a,*]

[a] *Department of Surgery, King's College Hospital, Denmark Hill, London SE5 9RS;*
[b] *Department of Histopathology, King's College Hospital, Denmark Hill, London SE5 9RS*

*(Received 25 August 1997; In final form 16 January 1998)*

**Pulmonary lymphangitis carcinomatosa is an unusal cause of death in a young adult. This case describes an apparently healthy young woman who presented with severe acute pancreatitis, which is a recognized complication of a choledochal cyst. Autopsy examination revealed advanced malignancy with poorly differentiated adenocarcinoma penetrating the wall of the choledochal cyst and metastatic adenocarcinoma in the lymph nodes, lungs and kidneys. This case emphasises the unusual presentation of a choledochal cyst with acute pancreatitis and the aggressive nature of malignancy associated with this congenital anomaly.**

*Keywords:* Choledochal cyst, pancreatitis, advanced malignancy

### INTRODUCTION

Choledochal cyst is a rare congenital anomaly. It is estimated to occur in 1:100 to 1:150,000 live births in western populations and is more common in females than males [1]. The incidence in Japanese populations is higher. The classical presentation is described as the triad of abdominal pain, jaundice and an abdominal mass [1], although few patients present initially with all three features. Acute pancreatitis [2, 3] and malignant change [3, 4] in the cyst wall are now recognized as complications, the incidence of carcinoma increasing with the age of the patient. Cysts diagnosed in the first decade of life have a 0.7% risk of developing malignant change and this rises to 15.5% in adults [4].

The following case is reported to describe malignant change in a choledochal cyst presenting with acute pancreatitis. It illustrates the rapid progression of carcinomatous change which may occur even in those patients who do not have a long clinical history.

*Correspondence to: Prof. of Hepatobiliary Surgery, King's College Hospital, Denmark Hill, London SE5 9RS.

### CASE REPORT

A 26 year old female presented as an emergency with a short history of abdominal pain and lumbar back pain. The serum amylase was 835 IU/l indicating a diagnosis of acute pancreatitis. Intermittent symptoms of upper abdominal pain and back pain had occured during the three months prior to admission. Liver function tests were normal. CT scan and ultrasound examination showed an enlarged and inflamed pancreas with free intra-peritoneal fluid and dilatation of the extrahepatic bile ducts typical of a choledochal cyst (Fig. 1). This episode of acute pancreatitis resolved with conservative management and the patient was discharged home to await further investigation.

Two weeks later abdominal pain and vomiting recurred. Examination showed a tender mass in the epigastrium and enlargement of the liver for 3 to 4 cm below the costal margin. Serum amylase was within normal limits but liver function tests showed a bilirubin of 102 umol/1 (*n* 3–20), alkaline phosphatase 784 IU/L (*n* 30–120), gamma glutamyl transferase 2015 IU/L (*n* 5–55) and asparate transferase 589 IU/L (*n* 10–50). A nuclide excretion scan (99 Tc hydroxy indole diacetic acid, HIDA) demonstrated no uptake by the liver and no biliary excretion. Treatment was commenced with analgesia, parenteral nutrition, intravenous H2 blockers, intravenous octreotide and nasogastric suction. Six days later an ERCP was attempted. There was distortion of the duodenum due to external compression and cannulation of the papilla failed. Clinical signs worsened and ascites developed in association with deteriorating liver function and severe peritoneal irritation. The amylase content of the ascites was only 19 IU/L. A laparotomy was performed because of the signs of peritonitis. The findings included: 1.2 l of ascites and inflammation of an enlarged pancreas. The mesentery of the small and large bowel was thickened, inflammed and adherent

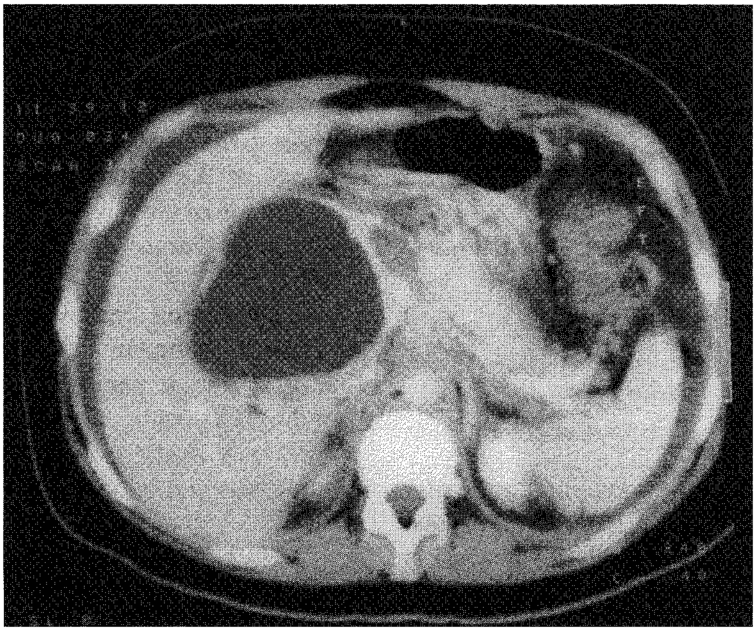

FIGURE 1 CT Scan demonstrating a large choledochal cyst, severe pancreatitis with peri-pancreatic inflammatory tissue and small volume ascites.

to the pancreas. The structures of the right hypochondrium were densely adherent forming a large friable inflammatory mass and the choledochal cyst could not be dissected safely. The gall bladder was drained *via* a cholecystostomy and there was no evidence of gall stones or biliary sludge. Peripancreatic drains were inserted. The inflammatory changes precluded a detailed assessment of the biliary tract and there was no macroscopic evidence of malignant change.

The first day after laparotomy was complicated by acute dyspneoa with an oxygen saturation of 7.5 kPa (*n* 10.67 – 13.33). The clinical suspicion of a pulmonary embolism was not confirmed on ventilation/perfusion scan. Clinical and biochemical improvement was seen over the next 10 days and a cholangiogram performed through the cholecystostomy tube confirmed the presence of a choledochal cyst. A repeat HIDA scan showed improved liver function, good excretion of bile through the cholecystostomy tube but no drainage into the duodenum, suggesting complete obstruction of the lower common bile duct. A second episode of respiratory distress was followed by a massive haematemesis. Tracheal intubation revealed a large quantity of blood in the trachea and larynx. The patient died the following day. The cause of death as determined by post mortem examination was cardiorespiratory failure secondary to aspiration and acute pancreatitis.

### HISTOPATHOLOGY

The presence of a choledochal cyst was confirmed at post mortem examination. It was described as a saccular diverticulum projecting from the right border of the supraduodenal common bile duct (Todani Classification type 11) [1]. Superiorly the cyst was continuous with the right and left hepatic ducts and inferiorly with the common bile duct. It measured 9 × 8 cm and was densely adherent to the pancreas and liver. The pancreas was enlarged and inflamed and extensive fat necrosis was present throughout the upper abdomen. There was no macroscopic evidence of malignant change.

Microscopically the mucosa of the choledochal cyst was autolysed and contained clusters of mucin containing tumour giant cells which infiltrated through the muscular wall of the cyst to the serosal surface (Fig. 2). In the region of the head of the pancreas the tumour infiltrated autonomic ganglia and nerve trunks. The tumour cells stained positive for mucin on PAS staining. The appearences were those of a poorly differentiated adenocarcinoma arising in the choledochal cyst and extending through the wall. Pulmonary lymphangitis carcinomatosa (Fig. 3) and metastatic adenocarcinoma to the lymph nodes and kidneys were also observed.

### DISCUSSION

Previous reviews have sugested that 60% of choledocal cysts present before 5 years of age [5], although a recent report suggests a changing pattern of presentation with more patients presenting as adults [6]. In a 17 year experience of 42 patients with choledochal cysts 31 were adults. In a further report of a 32 year experience with 13 patients 8 were adults [7].

Congenital dilatation of the bile duct is associated with an anomalous junction of the pancreaticobiliary ductal system in more than 70% of patients [8, 9]. An association with acute pancreatitis was first reported in 1957 [8] and many cases have now been described. Pancreatitis is not commonly associated with choledocholithiasis in these patients. However a post mortem examination in out patient did not identify an anomalous pancreatico-biliary junction or gall stones.

Choledochal cysts, particularly in adults, were originally treated with either drainage or bypass procedures. Approximately 40% of these patients developed late complications of recurrent

FIGURE 2 Infiltration of the cyst wall by malignant glands (H + E × 100). (See Color Plate I).

FIGURE 3 Pulmonary lymphangitis carcinomatosa (Trichrome stain × 100). (See Color Plate II).

jaundice and cholangitis requiring reoperation [2]. Now the treatment of choice is excision of the cyst and Roux-en-Y hepaticojejunostomy [2]. Excision of the entire cyst is necessary for biliary tract drainage and for removing the potential for malignant change in the cyst wall. In our case the tumour had penetrated the cyst wall to the serosa and invaded the lymphatics and perineural tissue.

The incidence of carcinoma in choledochal cysts is 5–35 times greater than in normal sized bile ducts [10] and the risk increases with age [4]. Malignant change is rarely found in children and there are only three reports of patients less than 18 years the youngest of whom was 12 years old [11]. Adenocarcinoma is the tumour most frequently associated with choledochal cysts but anaplastic, squamous cell and sarcomatous tumours have been described [11, 12]. Epithelial metaplasia is also common and it is postulated that the increased incidence of both metaplasia and carcinoma is secondary to chronic inflammation from presistent stasis of bile and reflux of bile and pancreatic juice into the cyst [4]. Reveille [10] postulated that both stasis and bacterial overgrowth, with the production of secondary bile acids are responsible for the pathological changes. The tumours associated with choledochal cysts are particularly aggressive. They are often clinically silent but are characterized by early lymphatic and perineural invasion [12] and the prognosis is very poor. Our patient showed this typical clinical course with no clinical symptoms prior to her presentation with acute pancreatitis. The findings of lymphangitis carcinomatosis and metastases to the kidneys at autopsy were not anticipated. In retrospect the diagnosis of diffuse malignancy may explain the very dense peripancreatic tissue found at laparotomy; but at that time the marked inflammatory component combined with the clinical condition and age of the patient did not suggest a diagnosis of cholangiocarcinoma.

This is the second documented case of pulmonary lymphangitis carcinomatosa associated with choledochal cyst [13]. The previous report described an 18 year old male who presented with acute respiratory failure. There was no evidence of pancreatitis. A chest X-ray revealed fine reticular densities and a massive right pleural effusion. The diagnosis of lymphangitis carcinomatosa metastatic from a choledochal cyst was made at autopsy.

This case illustrates two unusual complications of a choledochal cyst in a young patient. It emphasises the malignant potential of the abnormality and the poor prognosis in patients who develope a carcinoma within the dilated biliary tract.

### *References*

[1] Howard, E. R. (1991). Choledochal cysts. In Surgery of liver disease in children, edited by Howard, E. R., pp. 78–90. Oxford: Butterworth-Heinemann.

[2] Deizel, D. J., Rossi, R. L., Munson, L., Braasch, J. W. and Silverman, M. L. (1986). Management of bile duct cysts in adults. *Arch. Surg.*, **121**, 410–415.

[3] Hewitt, P. M., Krige, E. J., Bornman, P. C. and Terblanche, J. (1995). Choledochal cysts in adults. *Br. J. Surg.*, **82**, 382–385.

[4] Voyles, C. R., Smadja, C., Shands, C. and Blumgart, L. (1983). Carcinoma in choledochal cysts. An age-related incidence. *Arch. Surg.*, **118**, 986–988.

[5] Stringer, M. D., Dhawan, M., Davenport, M., Mieli-Vergani, G., Mowat, A. P. and Howard, E. R. (1995). Choledochal cysts: lessons from a 20 year experience. *Arch. Dis. Child.*, **73**, 528–531.

[6] Lipsett, P. A., Pitt, H. A., Colombani, P. M., Boitnott, J. K. and Cameron, J. L. (1994). Choledochal cyst disease. A changing pattern of presentation. *Ann. Surg.*, **220**, 644-652.

[7] Robertson, J. F. R. and Raine, P. A. M. (1988). Choledochal cyst: a 33-year review. *Br. J. Surg.*, **75**, 799–801.

[8] Okada, A., Nakamura, T., Higaki, J., Okumura, K., Kamata, S. and Oguchi, Y. (1990). Congenital dilatation of the bile duct in 100 instances and its relationship with anomalous junction. *Surg. Gyne. Obstet.*, **171**, 291–298.

[9] Rattner, D. W., Schapiro, R. H. and Warshaw, A. L. (1983). Abnormalities of the pancreatic and bile ducts in adult patients with choledochal cysts. *Arch. Surg.*, **118**, 1068–1073.
[10] Reveille, R. M., Van Steigmann, G. and Everson, G. T. (1990). Increased secondary bile acids in a choledochal cyst. Possible role in biliary metaplasia and carcinoma. *Gastroenterology*, **99**, 525–527.
[11] Iwai, N., Deguchi, E., Yanagihara, J., Iwai, M., Matsuo, H., Todo, S. *et al.* (1990). Cancer arising in a choledochal cyst in a 12-year old girl. *J. Ped. Surg.*, **25**, 1261–1263.
[12] Dowsett, J. F., Rode, J., Chandiramani, V. A. and Russell, R. C. G. (1991). Occult carcinoma in an adult choledochal cyst. *Postgrad. Med. J.*, **67**, 202–205.
[13] Chiang, Y. C., Lee, C. H. and Lin, P. Y. (1991). Pulmonary lymphangitis carcinomatosa due to adenocarcinoma arising from choledochal cyst: report of an autopsy case. *J. Formos Med. Assoc.*, **90**, 860–862.

### COMMENTARY

The authors describe a rate combination of complications in a young adult with a Todani type II choledochal cyst. Although cholangitis is the most common presenting symptom complex in adults, up to one-third of patients have associated pancreatitis [1,2]. A significant association between bile duct cysts and hepatobiliary malignancies is well established, the reported incidence of mitotic transformation ranging from 2.5 to 28% [3–5]. Malignancies associated with bile duct cysts arise not only within the cyst but also elsewhere within the liver or pancreaticobiliary tract and include cholangiocarcinoma or adenocarcinoma, adenocanthoma, squamous cell carcinoma, anaplastic carcinoma, bile duct sarcoma, hepatoma, pancreatic carcinoma and gallbladder carcinoma [2, 4, 5]. Only 57% of tumors are intracystic and may occur after cyst excision. Although 80% of carcinomas occur in patients with type I cysts, malignant change has also been described in Todani types II, III, IV and V cysts [2, 6]. The average age at presentation is 34 years, and 70% are women [2, 4].

Although the authors use the term "pulmonary lymphangitis carcinomatosa" which is widely used to refer to this particular pattern of tumor growth, the histologic basis of the interstitial widening seen both roentgenographically and grossly is seldom caused by pure lymphatic permeation; instead, a substantial (and occasionally the entire) part is caused by neoplastic infiltration and the fibroblastic reaction to it in the peribronchovascular and interlobular connective tissue outside lymphatic channels. Thus, the designation "lymphangitis carcinomatosa" is not strictly correct semantically; however, since these terms are deeply ingrained in our nomenclature and provided that the semantic inaccuracy is recognized, their continued use is regarded as acceptable [7]. Although virtually any neoplasm can result in disseminated pulmonary lymphatic and interstitial infiltration, (the rare occurrence of cholangiocarcinoma arising within a choledochal cyst in this instance) the most common primaries are the breast, stomach, pancreas and prostate. This case report adds to the growing body of evidence in the literature underscoring the necessity for a high index of suspicion of carinoma in adults with choledochal cysts.

### *References*

[1] Hewitt, P. M., Krige, J. E. J., Bornman, P. C. and Terblanche, J. (1995). Choledochal cysts in adults. *Br. J. Surg.*, **82**, 382–385.
[2] Rossi, R. L., Silverman, M. L., Braasch, J. W., Munson, J. L. and Remine, S. G. (1987). Carcinomas arising in cystic conditions of the bile ducts. A clinical and pathologic study. *Ann. Surg.*, **205**, 377–384.
[3] Dayton, M. T., Longmire, W. P. Jr. and Tompkins, R. K. (1983). Caroli's disease: a premalignant condition? *Am. J. Surg.*, **145**, 41–48.
[4] Voyles, C. R., Smadja, C., Shands, W. C. and Blumgart, L. H. (1983). Carcinoma in choledochal cysts: age-related incidence. *Arch. Surg.*, **118**, 986–988.
[5] Nagorney, D. M., McIlrath, D. C. and Adson, M. A. (1984). Choledochal cysts in adults: clinical management. *Surgery*, **96**, 656–663.

[6] Chaudhuri, P. K., Chaudhuri, B., Schuler, J. J. and Nyhus, L. M. (1982). Carcinoma associated with congenital cystic dilation of bile ducts. *Arch. Surg.*, **117**, 1349–1351.

[7] Fraser, R. G., Paré, J. A. P., Paré, P. D., Fraser, R. S. and Genereux, G. P. (1989). Diagnosis of Diseases of the chest. WB Saunders Company, Philadelphia, pp. 1146–1149.

JEJ Krige,
Surgical Gastroenterology,
Department of Surgery,
University of Cape Town Medical School,
Anzio Road,
Observatory 7925,
Cape Town,
South Africa.

